# Microfluidic Isolation of Disseminated Tumor Cells from the Bone Marrow of Breast Cancer Patients

**DOI:** 10.3390/ijms241813930

**Published:** 2023-09-11

**Authors:** Léa L. Volmer, Cansu E. Önder, Barbara Volz, Anjali R. Singh, Sara Y. Brucker, Tobias Engler, Andreas D. Hartkopf, André Koch

**Affiliations:** 1 Research Institute for Women’s Health, University of Tübingen, 72076 Tübingen, Germany; 2 Department of Women’s Health, University of Tübingen, 72076 Tübingen, Germany

**Keywords:** Parsortix, disseminated tumor cells (DTCs), breast cancer, microfluidic cell separation, liquid biopsy

## Abstract

Disseminated tumor cells (DTCs) in the bone marrow (BM) of breast cancer (BC) patients are putative precursors of metastatic disease, and their presence is associated with an adverse clinical outcome. To achieve the personalization of therapy on a clinical routine level, the characterization of DTCs and in vitro drug testing on DTCs are of great interest. Therefore, biobanking methods, as well as novel approaches to DTC isolation, need to be developed. In this study, we established a protocol for the biobanking of BM samples and evaluated a microfluidic-based separation system (Parsortix^®^) for the enrichment of cryopreserved DTCs. We were able to successfully isolate viable DTCs after the prior cryopreservation of BM samples. We calculated a significant increase of up to 90-fold in harvested DTCs with the proposed method compared to the current standard techniques, opening up new analysis possibilities for DTCs. Our advanced method further presents options for 3D DTC cultures, enabling the individualized testing of targeted therapies for BC patients. In conclusion, we present a novel approach for DTC enrichment, with possibilities for future clinical implications.

## 1. Introduction

Micro-metastasis plays a role in the prognosis of breast cancer (BC) patients; the presence of disseminated tumor cells (DTCs) in the bone marrow (BM) of the patients is associated with a poorer outcome [1], as well as with earlier locoregional and distant relapses [2,3,4]. BM can act as a niche for DTCs, where tumor cells can remain dormant, leading to disease recurrence after BC treatment [5,6]. An analysis of DTCs is therefore the key to understanding the mechanisms of metastasis in BC.

The molecular analysis of DTCs can offer insights into cancer propagation. For example, comparing the genetic profiles of DTCs to those of lymph node metastases has shown that different clones of the primary tumor can metastasize [7]. From a clinical point of view, it is relevant that distant metastases may have different biological profiles than the primary tumor [8]. In the future, the molecular characterization of initial DTCs sampled at the time of the initial diagnosis may provide not only prognostic information, but also information for therapeutic decisions regarding adjuvant treatment or in the case of disease recurrence.

To date, a patient’s BM is most commonly collected during breast surgery at the time of the initial diagnosis, so a possible recurrence of the disease at a later time is not yet known [9,10]. This emphasizes the need for DTC analysis, not only immediately at the time of the primary diagnosis, but also later, at the time of disease recurrence, especially if new diagnostic and therapeutic options are available at the time of recurrence. Therefore, the establishment of methods for the biobanking of DTCs and BM seems inevitable.

Most often, DTCs are enriched and detected according to the standard technique involving the cytokeratin immunostaining of cytospins from isolated BM mononuclear cells [11]. For later analysis, these cytospins can be preserved at −80 °C. The numbers of DTCs per patient that are available for analysis then range from single cells to, very rarely, double-digit numbers [12]. Accordingly, only a few cells can be manually harvested via microdissection or micromanipulation [13]. In order to compensate for the very limited number of cells available, methods such as single-cell sequencing must be applied [7]. Other DTC detection and enrichment methods also rely on the DTCs’ antibody expression, for example, EpCAM or HER2 [14,15]. However, BC cells can undergo epithelial-to-mesenchymal transition [16,17,18], thereby reducing or losing the expression of epitopes used for DTC enrichment and detection. Aside from the problem of the reduced enrichment of DTCs, this can additionally lead to a false-negative DTC detection.

To overcome these challenges, new enrichment methods and devices based on microfluidic cell separation were developed. Some devices are solely based on cell size, while others additionally rely on cell-surface markers or the electrophysical properties of cells [19,20,21].

The Parsortix platform is an epitope-independent, microfluidic-based filtration system. Studies using Parsortix have already been able to successfully enrich and analyze circulating tumor cells (CTCs) from peripheral blood [22,23,24]. More recently, it was shown that using Parsortix may also be a feasible method to enrich and characterize DTCs from fresh bone marrow [25]. Another important feature of Parsortix microfluidic cell separation is that the cells are viable once harvested, theoretically opening the possibility of culturing isolated DTCs. By establishing methods of culturing DTCs, clinically relevant drug assays can be performed.

In light of the above challenges, the aim of this study is to optimize the detection and biobanking methodology of BM DTCs. For this purpose, an epitope-independent method using Parsortix and a subsequent protocol for permanent preservation was established and compared with standard DTC detection methods.

## 2. Results

Cytokeratin staining, full laboratory processing information, and clinical data were available for 361 patients. Supplementary whole bone marrow was either processed immediately or cryopreserved for 206 of these patients. See the supplementary data for the clinical data and the characteristics of patients whose BM was processed with Parsortix or cryopreserved (Table S1).

**Figure 1:**
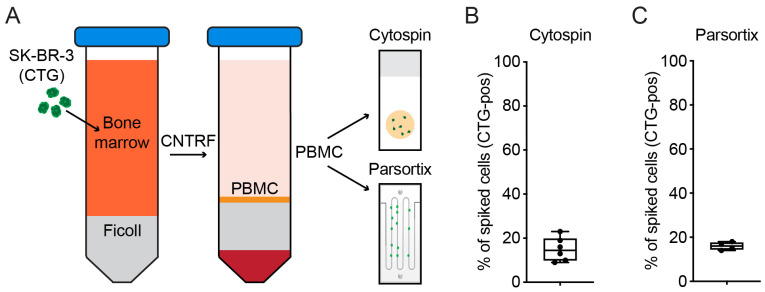
Spiking experiments with Ficoll gradient enrichment of PBMC. (**A**) Workflow of experiment. SK-BR-3 cells (Cell Tracker Green labeled) were spiked in whole BM followed by PBMC isolation. The PBMC fraction was then subjected to either cytospinning or Parsortix enrichment. CTG-positive cells were then counted on cytospins (**B**) or in the Parsortix cassette (**C**). (**B**) Box plot for enumerated CTG-positive cells on cytospins normalized to the total number of spiked cells (*n* = 6). (**C**) Box plot for enumerated CTG-positive cells present in the Parsortix harvest cassette normalized to the total number of spiked cells (*n* = 4).

The standard method of DTC detection [11] starts with a density centrifugation step to enrich the mononuclear cells (PBMCs) and DTCs in the same fraction with a subsequent cytospinning of this fraction onto glass slides. After an immunostaining step (e.g., cytokeratin), the DTCs are enumerated. However, the PBMC fraction often contains such a high number of cells that more than a double-digit number of cytospins would need to be prepared to be able to detect all the DTCs present in the sample. According to the consensus recommendation, only two cytospins are stained and evaluated for the detection of DTCs [11]. To reduce the amount of actual PBMCs and increase the fraction of DTCs, we wanted to evaluate if a supplementary step involving microfluidic cell enrichment after the density centrifugation of mononuclear cells would be beneficial. For this purpose, fresh BM samples were spiked with fluorescently labeled (Celltracker Green CMFDA—“CTG”) SK-BR-3 cells. The SK-BR-3 cell line was chosen for this study due to its importance as a breast cancer research model. These cells display an epithelial morphology, express cytokeratins, and show an overexpression of HER2 [26]. Due to these characteristics, they also serve as positive controls in routine DTC detection (Supplementary Figure S1). For all spiking experiments, SK-BR-3 cells were added to the BM at a similar density to the expected numbers of the patients’ DTC samples. After standard density centrifugation, the PBMC fraction was halved and was either used directly for cytospin preparation or additionally processed via microfluidic cell separation (Figure 1A). The fluorescently labeled cells on all of the cytospins were counted and compared with the number of fluorescent cells being captured in the cell separation cassette (Figure 1A). Both processing methods with an initial density centrifugation step yielded similar results: with the standard processing method alone, a mean of 14.9% (Q1–Q3: 10.8–15.6%; median 14.2%) of spiked cells were present on the cytospins (Figure 1B), whereas with the Parsortix cell enrichment step, a mean of 15.7% (Q1–Q3: 14.5–17.1%; median 15.6%) of spiked cells were present in the separation cassette (Figure 1C). Therefore, 84.3–85.1% of spiked cells were lost during the standard BM processing (PBMC isolation) regardless of the enumeration method used.

**Figure 2:**
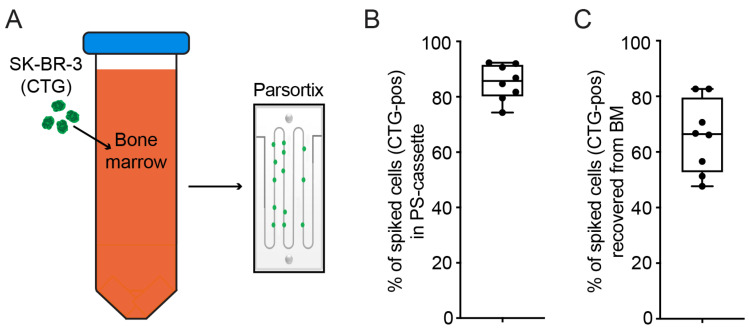
Spiking experiments with microfluidic separation of whole BM. (**A**) Workflow of experiment. SK-BR-3 cells (Cell Tracker Green labeled) were spiked in whole BM followed by Parsortix enrichment. Cells were then enumerated before harvest (**B**) and after harvest (**C**). (**B**) Box plot of CTG-positive cells captured in the Parsortix cassette normalized to the total number of spiked cells. (**C**) Box plot of CTG-positive cells (normalized to the total number of spiked cells) recovered from whole BM after Parsortix enrichment. A total of eight experiments were performed.

As we suspected that the PBMC isolation step was the limiting factor, we wanted to evaluate if microfluidic separation using whole BM instead of PBMCs would result in higher numbers of harvested DTCs. To this end, CTG-labeled SK-BR-3 cells were spiked into fresh whole BM prior to processing with Parsortix (Figure 2A; see the Methods section for details). In the first step, the Parsortix system enriches cells in a separation cassette. The enriched cells can then either be used directly in the cassette (e.g., for immunofluorescence staining) or washed out of the cassette for downstream analysis. We therefore first evaluated how many cells can be captured in the cassette (Figure 2B), and second, we evaluated how many cells can be recovered from the cassette (Figure 2C). When using the Parsortix method with whole BM, a mean of 85.2% (Q1–Q3: 81.1–91.0%; median 85.7%) of spiked cells could be captured in the separation cassette (Figure 2B). By counting the number of cells retained in the enrichment cassettes after the final harvest step, we were able to calculate the overall percentage of cells recovered when using Parsortix (Figure 2C). On average, 65.6% (Q1–Q3: 55.3–73.7%; median 66.4%) of spiked SK-BR-3 cells could be enriched using this method (Figure 2C), resulting in an average cell loss of 34.4% during the entire process. Therefore, when using the Parsortix cell separation method with whole BM, a four-fold increase in the number of harvested cells could be achieved compared to the standard method of using a density centrifugation step to obtain PBMCs (compare Figure 1B and Figure 2C; 14.9% vs. 65.6%, *p* = 0.0002).

### 2.1. Cryopreservation of BM Enables Biobanking of DTCs

Currently, BM is collected at the time of the first diagnosis and immediately processed into cytospins. The biobanking of sufficient numbers of DTCs with the possibility of a later cell analysis at the time of a later disease recurrence or metastasis is therefore still lacking in routine BM handling. To establish a biobanking method for BM derived from BC patients, we developed a protocol outlining the cryopreservation, thawing, and subsequent processing of patient samples. Starting from whole BM, we were able to cryopreserve samples from *n* = 206 patients. To quantify cell loss during cryopreservation and thawing, three separate whole BM samples (5–10 mL) from patients were spiked with CTG-labeled SK-BR-3 cells, processed for cryopreservation, and stored at −196 °C for at least 24 h. The samples were then thawed according to our established protocol (see Methods), and the cells were spun onto cytospins and counted as described above. A mean of 60.7% (50.9–72.6%) of the original spiked cells were enumerated (Table 1). As our primary objective was to biobank the BM for comprehensive future analyses, we stopped the thawing and processing of additional samples after this proof-of-concept evaluation.

### 2.2. Detection of DTCs Is Possible after Parsortix Cell Separation

**Figure 3:**
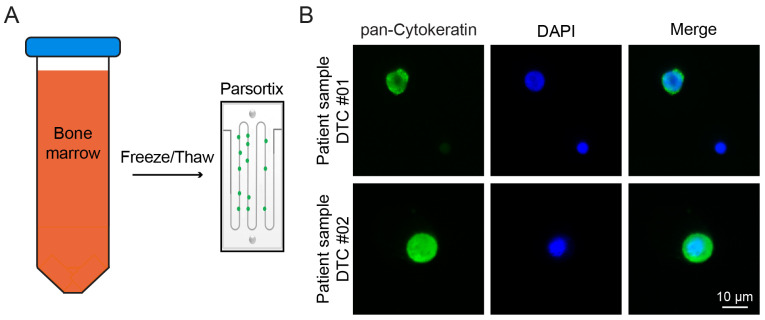
Recovery of patient DTCs from cryopreserved BM. (**A**) Workflow of experiment; BM samples were cryopreserved and subsequently thawed for Parsortix enrichment (see Methods for details). (**B**) Immunofluorescence images of harvested cells. DTCs can be identified as pan-CK+/DAPI+, and mononuclear cells can be identified as pan-CK-/DAPI+.

To further investigate the feasibility of whole BM processing in real laboratory conditions with real patient samples, we evaluated the ability to detect DTCs in patient samples after cryopreservation and cell separation using Parsortix. Four cryopreserved samples were used, in which DTCs were detected using the standard method. These four samples were classified as CK 1+ or 2+ using the standard detection method (see Supplementary Table S2 for CK classification). The samples were thawed according to the described protocol. Microfluidic cell separation was performed, and the resulting harvest was stained for cytokeratin (see Methods) and analyzed via fluorescence microscopy (Figure 3). Based on the results of our previous spiking experiments (Figure 2C, Table 1), we calculated an estimated number of DTCs to be expected in the final fluorescence microscopy count (number of expected cells after Parsortix cell separation = number of cells after standard method × total sample volume × percentage of harvested cells in Parsortix spiking experiment). DTCs were detected in each of the patient samples (see Figure 3B for examples), demonstrating a 100% detection rate for DTC positivity when using the described microfluidic cell separation method. DTCs were present at high densities (see Table 2), therefore confirming a higher yield after the microfluidic separation of whole BM compared to the standard method.

### 2.3. Microfluidic Cell Separation Displays Potential for Isolating High Numbers of DTCs from Patient Samples

Having previously observed a four-fold increase in the number of cells harvested with Parsortix cell separation in our spiking experiments (Figure 1B compared to Figure 2C), we sought to extrapolate these findings to the actual patient data. Therefore, we evaluated how cell separation using Parsortix instead of the standard method would affect the number of retrievable DTCs for downstream analysis from routinely collected BM. To this end, the potential number of isolated cells was calculated based on the data from the standard method and the harvest percentages from the described spiking experiments (see Table 1/Figure 2C). Table 3 provides an overview of the categorization of our DTC-positive patient samples according to the number of cells detected in a total of 3.0 × 10^6^ cells using the standard method. The samples with >10 DTC per 3.0 × 10^6^ of total cells (*n* = 2) were not included. Table 4 compares the median number of cells that would be obtained from the patient samples using each method. For a CK 1+ sample, after standard processing, a maximum of 3 cells would potentially be available for a downstream analysis, whereas an average of 252 cells would be available when processing the fresh whole BM sample with Parsortix. For a CK 2+ sample, a maximum of 7 cells would be used after standard processing compared to an average of 651 cells (mean) after Parsortix cell separation. Finally, for a CK 3+ sample, a maximum of 10 cells would be available after standard processing compared to 880 cells (mean) after Parsortix cell separation. Supplementary Figure S2 shows the expected amount of DTCs that would be obtained from fresh whole BM after Parsortix cell separation for all patient samples (source data; see Table S3).

### 2.4. Parsortix Cell Separation Enables 3D Culture of Cancer Cells after BM Cryopreservation

**Figure 4:**
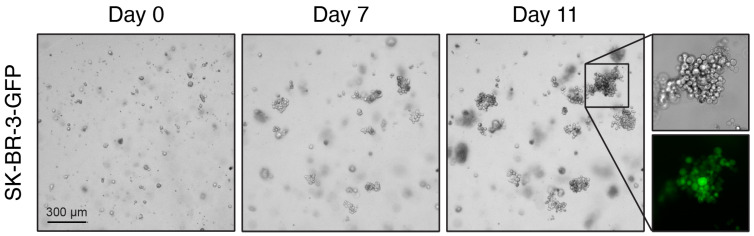
Cell separation with Parsortix enables 3D culture of SK-BR-3 cells after BM cryopreservation. Brightfield images (days 0, 7, and 11 of culture) of SK-BR-3 cells (stably expressing GFP) cultured in BME after spiking into BM sample, cryopreservation, and separation with Parsortix. Insert (day 11) shows a grape-like organoid as brightfield and GFP. Scale bar: 300 μm.

As cryopreservation and the harvesting of large numbers of DTCs from BM appears to be feasible, we wanted to explore the possibilities for downstream cell characterization and analysis offered by microfluidic cell separation from whole BM. Knowing that this method could potentially yield high numbers of DTCs (Table 4 and Supplementary Figure S2), we wanted to assess whether performing cell cultures of harvested DTCs after microfluidic cell separation might be feasible. As patient-derived tumor cells have been shown to exhibit improved proliferation in 3D cultures, we decided to evaluate the possibility of generating organoid cultures of breast cancer cells. To this end, SK-BR-3-GFP cells were spiked into fresh whole BM prior to cryopreservation, followed by cell separation using Parsortix after thawing. The harvested cells were plated in extracellular matrix surrogate basement membrane extract, and their ability to form 3D organoids was evaluated. Indeed, organoids, exhibiting the characteristic SK-BR-3 grape-like growth morphology [27], formed and proliferated for at least 21 days (Figure 4).

## 3. Discussion

DTCs in BM are associated with disease recurrence even after the full completion of guideline-conform therapy [9,28]. Studying DTCs is of paramount importance in understanding the mechanisms underlying tumor cell dissemination in breast cancer [29]. However, the current research on DTCs has primarily focused on their detection and enrichment based on the epitopes expressed on these cells [11], resulting in the isolation of a limited number of viable cells for further characterization. In this study, we tested the microfluidic cell separation system Parsortix for its ability to overcome these limitations in translational DTC research by isolating large numbers of DTCs after biobanking via cryopreservation and obtaining viable cells.

Our protocol for the cryopreservation of DTCs in BM lays the foundation for new possibilities in the study of DTCs. We have shown that the biobanking of fresh whole BM samples is feasible for routine clinical practice, as is cell separation using Parsortix after the thawing of cryopreserved samples. As a proof of principle, the epitope-based detection of DTCs after the thawing of the samples was not affected by cryopreservation, demonstrating that standard detection methods can still be used. In an analogy to CTC research, it would then be possible to perform an in-depth characterization of DTCs even after the cryopreservation of patient samples [30,31]. This would allow for sample selection at a later time point according to disease recurrence, which is not always predictable by clinical or biological risk factors [32,33,34,35].

In our study, an average of 65.6% of spiked cells could be enumerated after the cell separation of BM with Parsortix and subsequent harvesting (Figure 2C). These results differ from the 26% yield obtained in another study using DTCs from BM [25]. However, the results of the present study are in line with the harvest rates of 72.8% of melanoma DTCs in lymph nodes [36] and 54–69% of CTCs in other studies [22,23,37]. As microfluidic cell separation methods are not an established field of research, the differences between cell separation protocols, particularly in instrument settings, may lead to variable results. Other methods of the microfluidic separation of cancer cells based on mechanical cell properties have been described [19,38,39,40,41,42,43]. A study using the ClearCell^®^ FX1 device (Biolidics Limited, Mapex, Singapore) was able to obtain viable CTCs from non-small-cell lung cancer and establish xenograft models [44]. However, a direct comparison in terms of the cell number or viability is not possible.

Overall, this new method results in much higher DTC yields than standard cryopreservation and separation approaches. To illustrate the practicality of this new method, for most DTC-positive samples, a median of 204 cells could be harvested from a maximum of two cryopreserved vials (total volume 3 mL) after thawing and cell separation with Parsortix (Figure S2). To obtain a similar number of cells using the standard detection method, a total number of at least 30 cytospins per sample would have to be preserved, stained, and microdissected for DTC isolation. This is a huge reduction in both the research material and workload.

Epithelial-to-mesenchymal transition is thought to facilitate the tumor cell dissemination in breast cancer [45]. During this process, DTCs lose the expression of epitopes that are classically used for detection and are therefore not amenable to standard isolation methods [46,47]. In contrast to standard DTC isolation, Parsortix’s epitope-independent method may overcome this difficulty. However, further experiments are required to fully validate epitope-independent cell isolation.

A potential disadvantage of isolating cells based on their size is that BM mononuclear cells are larger in diameter than peripheral blood cells and are therefore also captured during the separation process. This may result in a lower purity of DTCs than what is achieved during the isolation of CTCs, but cell characterization via RNA analysis is still possible [25]. For single-cell analysis, BM cell depletion steps may be required to bypass DTC identification via staining and morphological characteristics.

Even with this new technique, challenges in DTC isolation and characterization remain. For example, the presented method is time-consuming, with a processing time of about 100 min per sample and without the possibility of separating more than one sample at a time. The pre-selection of samples and technical advances with microfluidic devices may soon provide solutions to this aspect.

A very strong point in the use of this microfluidic-based protocol is that the cells harvested after separation are viable. It has already been shown that 2D cultures of spiked DTCs in lymph node cell suspension or of CTCs from blood samples are feasible [36,48]. We were able to establish organoid cultures of breast cancer cell lines spiked into BM after cell separation using Parsortix. In this study, cell numbers close to those that could be expected in the patient samples were used, demonstrating that 3D cultures of DTCs derived from patient samples may be feasible. Organoids are now established models for various cancer entities, offering new opportunities for the in vitro drug testing of targeted therapies [49]. As a result, the 3D culture of DTCs may contribute to understanding the mechanisms of disease recurrence and metastasis in BC [50], as well as those of resistance to certain treatments. However, further experiments are needed to validate the feasibility of 3D culture compared with real patient samples.

## 4. Materials and Methods

### 4.1. Ethic Statement

Bone marrow aspirates were obtained from BC patients (Clinical stages I–IV) during surgery at the University Women’s Hospital in Tübingen. The study was conducted in accordance with protocols approved by the Ethics Committee of the Eberhard Karls University of Tübingen (reference numbers 528/2019BO2 and 040/2023BO2), and complies with all relevant ethical regulations for research involving human participants. BM samples were obtained via unilateral BM aspiration from the anterior superior spina iliaca crest into heparin-containing tubes and processed within 24 h after aspiration. Written informed consent was obtained from all the participants. Clinical data and relevant information on BM processing were collected from 206 BC patients.

### 4.2. Cell Culture and Reagents

SK-BR-3 BC cell line utilized in the present study was obtained from ATCC. Cells were cultured in DMEM (Dulbecco’s modified eagle medium) supplemented with 10% FBS (fetal bovine serum), 50 µg/mL penicillin/streptomycin, and 2 mM L-glutamine (all reagents were purchased from Thermo Fisher Scientific, Waltham, MA, USA). Cells were incubated at 37 °C in a humidified atmosphere containing 5% CO_2_. For routine passaging, cells were washed with 1× DPBS (Dulbecco’s phosphate-buffered saline, Thermo Fisher Scientific, MA, USA) and treated with Trypsin-EDTA 0.05% (Sigma Aldrich, St. Louis, MO, USA) as recommended by ATCC product sheets.

### 4.3. Standard Method of Bone Marrow Preparation and Detection of Disseminated Tumor Cells

Mononuclear cells from the BM were isolated via density centrifugation (Ficoll, 1.077 g/mL, Biochrom, Berlin, Germany). If necessary, red blood cell lysis (RBC) was performed (buffer: 155 mM NH_4_Cl + 10 mM KHCO_3_ + 100 µM Na_2_EDTA in H_2_O; pH: 7.4). Concentration of mononuclear cells was determined using a Sysmex hemocytometer (XP-300™ Automated Hematology Analyzer, Sysmex Europe SE, Norderstedt, Germany). An amount of 1.5 × 10^6^ mononuclear cells were spun down onto a glass slide (Cytocentrifuge, Hettich, Tuttlingen, Germany), and fixed in 4% formalin. Immunocytochemistry (ICC) was performed on a DAKO Autostainer (DAKO, Glostrup, Denmark) using mouse monoclonal antibody directed against keratin 8/18 (Clone: 5D3 Thermo Fisher Scientific, Fremont, CA, USA). Two slides per patient were evaluated according to the consensus recommendations for standardized tumor cell detection [11]. DTC positivity was defined as at least one cytokeratin-positive cell with abnormal cell morphology per 3.0 × 10^6^ cells. When tumor cells were detected, a standardized classification was applied (see Table S2).

### 4.4. Bone Marrow Preparation for Cryopreservation and Microfluidic Cell Separation

BM aspirates were mixed with PBS at a ratio of 1:3 (BM:PBS), filtered through a 100 µm cell strainer (for removal of bone trabeculae), and centrifuged at 478× *g* for 10 min. After centrifugation, the supernatant was discarded, and the cell pellet was processed further as described below.

### 4.5. Epitope-Independent Cell Separation

Isolation of DTCs was performed using the Parsortix PR1 automated microfluidic system (Angle North America, Philadelphia, PA, USA). This microfluidic cell separation platform was previously described in [23]. A disposable 6.5 µm gap size separation cassette (ANGLE PLC, Surrey, UK) was used and primed with 100% ethanol, followed by PBS, according to the manufacturer’s instructions. For direct cell separation, the cell pellet obtained as described above was resuspended in 10 mL PBS and transferred to a VacuTainer tube containing 1.8 mg/mL K2EDTA (Becton Dickinson, New York, NY, USA). For cryopreserved samples, thawed cells were resuspended in PBS and transferred to a VacuTainer tube containing 1.8 mg/mL K2EDTA. Cell separation was performed using the PX2_S99F instrument protocol according to the manufacturer’s manual. After cell separation, the captured cells were first harvested with 200 µL PBS, followed by a second 1000 µL PBS reverse flow using the PX2_H instrument protocol according to the manufacturer’s manual.

### 4.6. Cryopreservation and Thawing Procedure of Bone Marrow Cells

BM preparation was performed as described above. The resulting cell pellet was resuspended in RBC lysis buffer (buffer: 155 mM NH_4_Cl + 10 mM KHCO_3_ + 100 µM Na_2_EDTA in H_2_O; pH: 7.4) and centrifuged at 478× *g* for 10 min. After centrifugation, cells were resuspended in freezing medium (Recovery™ Cell Culture Freezing Medium, Thermo Fisher Scientific, Fremont, USA), transferred to cryovials, and placed in freezing containers (CoolCell™ LX, Corning, NY, USA) in a −80 °C freezer for at least 4 h before long-term storage in liquid nitrogen (−196 °C).

Samples were thawed by immersing the cryovials in a 37 °C water bath for 30 s. An amount of 100 µL of 10 mg/mL DNase I (Thermo Fisher Scientific, MA, USA) solution was immediately added to the cryovial, followed by 1000 µL of medium (DMEM + 10% FBS), both pre-cooled at 4 °C. The cell suspension was transferred to a tube containing 15 mL of pre-cooled medium (DMEM + 10% FBS) and centrifuged at 478× *g* for 10 min. The number of cells was determined using a Sysmex hemocytometer (XP-300™ Automated Hematology Analyzer, Sysmex Europe SE, Norderstedt, Germany). The cell pellet was resuspended in DMEM + 10% FBS. For cell separation using Parsortix, this suspension was transferred to a VacuTainer tube containing 1.8 mg/mL K2EDTA.

### 4.7. Evaluation of Cell Recovery after Separation Using Spiked Cells

For spiking experiments, SK-BR-3 cells were fluorescently labeled with CellTracker™ Green CMFDA (5-Chlormethylfluoresceindiacetat, Thermo Fisher Scientific, MA, USA) according to the manufacturer’s protocol. CellTracker Green was dissolved in DMSO and diluted in DMEM (Thermo Fisher Scientific, Waltham, MA, USA) to a final working concentration of 1 µM. Cells were incubated in CellTracker working solution for 45 min; then, the medium was removed and replaced with DMEM + 10% FBS (both reagents from Thermo Fisher Scientific, Waltham, MA, USA) for a second incubation of 30 min. Cells were harvested via trypsinization, counted using a Bio-Rad TC20 cell counter, fixed in 4% formalin solution, and stored in a non-transparent tube. A total of 125–900 cells per BM sample were added into 5–10 mL of fresh whole bone marrow. For each spiking experiment, both the CTG labeling efficiency and the number of spiked cells were checked by adding the same volume of spiked cells to three individual wells of a 48-well plate, followed by cell counting (GFP and brightfield) using a fluorescence microscope (EVOS M7000; Thermo Fisher Scientific, MA, USA). CTG labeling efficiency was 100% for each experiment. Samples were then processed with Parsortix as described above. Harvested cells were spun down onto a glass slide (Cytocentrifuge, Hettich, Tuttlingen, Germany) and fixed in 4% formalin. The resulting cytospins were mounted with ProLong™ Diamond Antifade Mountant with DAPI (Thermo Fisher Scientific, MA, USA). Slides were evaluated using an EVOS M7000 microscope at 10× and 20× magnification. Cells positive for Celltracker Green and DAPI were counted.

### 4.8. Cell Detection via Immunofluorescence

Patient samples were cryopreserved and thawed as described above. After centrifugation, the pellet was resuspended in 8 mL DPBS + 0.1% BSA (Bovine serum albumin, Thermo Fisher Scientific, MA, USA) and then transferred to a 10 mL VacuTainer tube containing 1.8 mg/mL K2EDTA. Cell separation was performed using Parsortix and a disposable 6.5 µm gap size separation cassette. After cell separation, the captured cells were harvested using a 200 µL + 1000 µL PBS reverse flow. The harvested cell suspension was spun down onto a 240 mm^2^ coverslip (cytocentrifuge, Hettich, Tuttlingen, Germany) and then fixed in 4% formalin. Each of the obtained cytospins was stained with an FITC-conjugated mouse monoclonal antibody against pan-cytokeratin (Pan-Cytokeratin (C-11)—FITC, Thermo Fisher Scientific, MA, USA). Coverslips were mounted on glass slides using ProLong™ Diamond Antifade Mountant with DAPI (Thermo Fisher Scientific, MA, USA). Cells determined to be positive for FITC and DAPI via fluorescence microscopy were defined as DTCs.

### 4.9. Three-Dimensional Culture of SK-BR-3 Cells after Microfluidic Cell Separation

SK-BR-3 cells stably expressing GFP (*n* = 6 × 10^4^ cells) were added to 5 mL of fresh whole bone marrow. Samples were prepared for microfluidic cell separation as described above. The cell suspension was separated with Parsortix as described above. The harvested cell suspension (30% ratio) was mixed with extracellular matrix surrogate basement membrane extract (70% ratio) (Corning^®^ Matrigel^®^ Basement Membrane Matrix, Radnor, PA, USA) and then plated as domes (*n* = 6) in a 48-well microplate (Corning Incorporated, NY, USA). Briefly, 20 µL drops were formed per well; then, the plate was inverted and placed in an incubator (37 °C, 5% CO_2_) for solidification. Afterwards, DMEM supplemented with 10% FBS (fetal bovine serum), 50 µg/mL penicillin/streptomycin, and 2 mM L-glutamine (all reagents from Thermo Fisher Scientific, MA, USA) was added to the domes, and the cells were cultured under standard conditions in humidified incubators at 37 °C with 5% CO_2_. The culture medium was renewed every 3 days.

### 4.10. Imaging

Imaging was performed on the EVOS M7000 Imaging system (Thermo Fisher Scientific, MA, USA).

### 4.11. Statistical Analysis

For statistical comparison of the results of spiking experiments, Student’s *t*-test was performed. All descriptive statistics and statistical analyses were performed using JMP15 (SAS^®^).

## 5. Conclusions

Our results demonstrate that the biobanking of BM from BC patients with a subsequent microfluidic isolation of DTCs is not only feasible, but can be successfully implemented in a clinical setting. The isolated DTCs were abundant and viable, which greatly facilitates their characterization and culture, which cannot be achieved with the current standard method for DTC detection. Although this method may not become the standard procedure for high-throughput diagnostics, it has several advantages over the standard DTC detection method and opens up new avenues for DTC research.

## Figures and Tables

**Table 1:** Cell recovery in spiking experiment of SK-BR-3 cells in whole BM after cryopreservation and thawing steps.

	Experiment 1	Experiment 2	Experiment 3
Spiked cells	124	163	188
Harvested cells	90	83	110
% Harvested cells	72.6%	50.9%	58.5%

**Table 2:** Characteristics of cryopreserved and separated samples with respect to the presence of DTCs.

Sample ID	CK-Classification *	Expected Number of DTCs **	Number of DTCs Detected
DTC #01	1+	160	125
DTC #02	2+	100	87
DTC #03	2+	50	83
DTC #04	2+	20	17

* See Table S2. ** Number of expected cells after Parsortix cell separation = number of cells after standard method × total sample volume × percentage of harvested cells in Parsortix spiking experiment.

**Table 3:** Repartition of patient samples following the CK classification.

Sample Classification	No. of Patient Samples (%)	Number of Tumor Cells in 3.0 × 10^6^ Total Cells
**DTC positive**	361 (100.0)	≥1
**CK 1+**	306 (84.7)	1–3
**CK 2+**	46 (12.7)	4–7
**CK 3+**	9 (2.5)	8–10

**Table 4:** Number of cells expected to be isolated from patient samples after standard processing or Parsortix cell separation.

Sample Classification	Median Number of Cells Expected after Standard Method (n)	Median Number of Cells Expected after Parsortix *, 95% CI
**CK 1+**	2	204 (83–794)
**CK 2+**	5.5	528 (190–2073)
**CK 3+**	9	737 (405–1925)

* *n* = 15 samples with outlying sample volume/cell density outside of 95% CI were excluded from calculation (see Table S3).

## Data Availability

The data presented in this study are available upon request from the corresponding authors.

## Supplementary Materials

The following supporting information can be downloaded at https://www.mdpi.com/article/10.3390/ijms241813930/s1.
