# Supplementary material for: Microfluidic Isolation of Disseminated Tumor Cells from the Bone Marrow of Breast Cancer Patients
Source: Int J Mol Sci. 2023 Sep 11;24(18):13930. doi: 10.3390/ijms241813930 (PMC10531360; doi:10.3390/ijms241813930)
Supplement: Supplementary file 1 [file ijms-24-13930-s001.zip › ijms-2573892-supplementary.pdf]

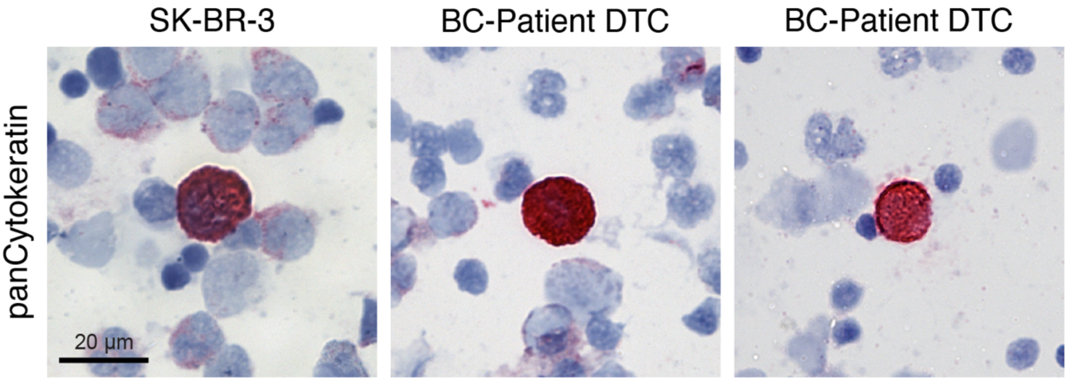

Supplementary Figure S1: Immunocytochemistry staining of bone marrow PBMCs. Bone marrow PBMC cytospins from breast cancer patients stained for panCytokeratin. On the left, SK-BR-3 cells were spiked into the sample beforehand. The middle and right panels show real patient DTCs. Scale bar = 20 µm.

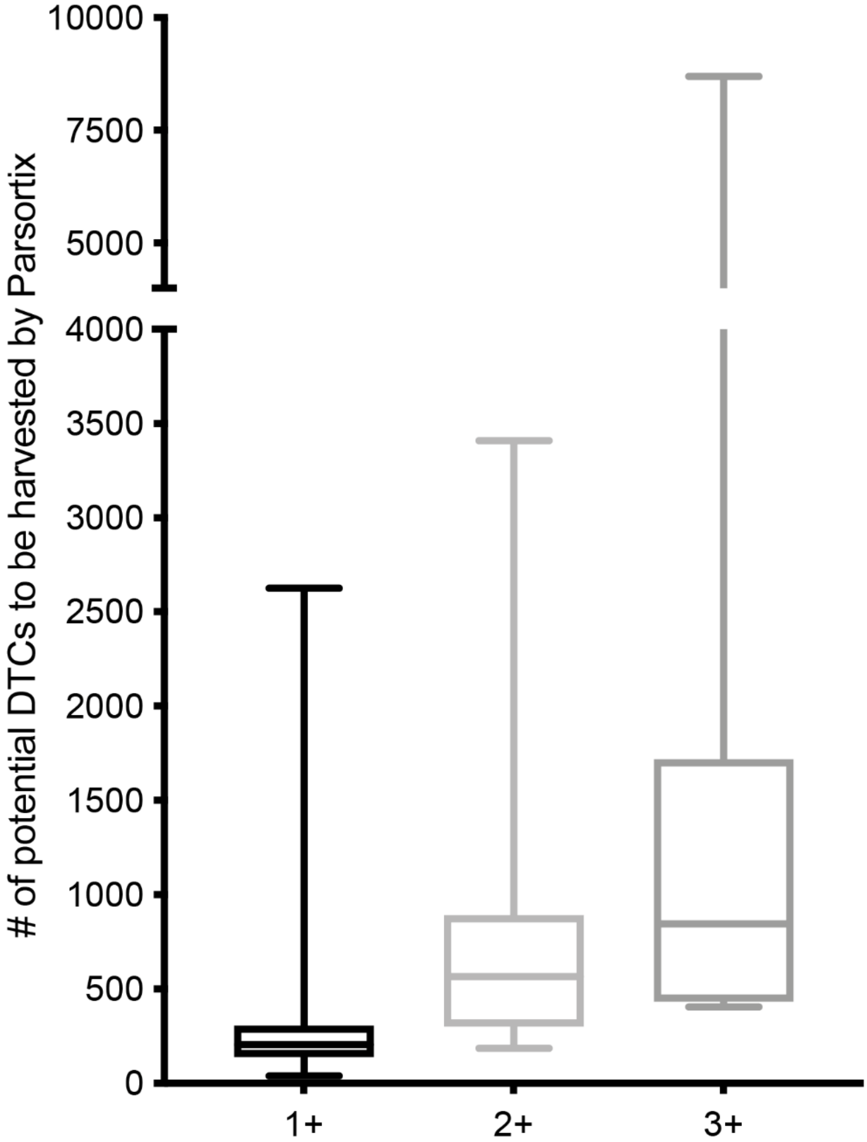

Supplementary Figure S2: Box plot of the number of DTCs potentially expected after Parsortix cell separation of whole BM from real patient samples. See Table S3 for source data.

Supplementary Table S1: Patient characteristics using a standard DTC detection method in relation to DTC positivity

	Total n	DTC positive* n (%)	p-value
Total	206	39 (18.9)	
Treatment prior to BMA			
Neoadjuvant chemotherapy	46	17 (37.0)	
No prior chemotherapy	160	22 (13.8)	0.001
Mean age (years)	59.3	58.3	0.690
Menopausal status			
premenopausal	53	13 (24.5)	
postmenopausal	153	26 (17.0)	0.237
Histology			
non-special type	156	33 (21.2)	
DCIS	17	2 (11.8)	
other subtype	33	4 (12.1)	0.327
Grading			
G1-2	119	18 (15.1)	
G3	65	19 (29.2)	0.025
Initial tumor size			
T0-1	113	16 (14.2)	
T2-4	23	23 (24.7)	0.054
Initial nodal status			
N0	121	19 (15.7)	
N1-3	71	17 (23.9)	0.163
Metastastic disease			
yes (M1)	1	0	
no (M0)	203	38 (18.7)	0.520
Subtype			
Triple negative	19	2 (10.5)	
Luminal-like	132	24 (18.2)	
Her2 positive	35	9 (25.7)	0.364

* as per routine cytospins/cytokeratin-staining method

Supplementary Table S2: Classification of samples with disseminated tumor cells (standard method)

Classification	number of tumor cells in total 3.0 x 10^6 cells
CK 1+	1-3
CK 2+	4-7
CK 3+	8-10

Supplementary Table S3: Sample characteristics of real patient samples and potential expected number of DTCs

A) Expected number of cells after standard isolation method (median), B) Expected number of cells after Parsortix isolation using whole BM. The value in B is being calculated from the total number of possible cytospins (not shown), a factor of '5' to account for the loss of DTCs during PBMC isolation (see Figure 1) and the harvest capacity (0.6×) of the Parsortix method (Figure 2C). Example for patient #8: 9 DTCs × 32 additional cytospins × 5 × 0.6 = 864 DTCs possible during Parsortix method. Data used as source for Supplementary Figure S2.

DTC patient ID	DTC-classification	A	B
#5	2+	5,5	219
#6	3+	9	432
#7	1+	2,5	90
#8	3+	9	864
#9	3+	9	405
#10	3+	9	432
#11	1+	2,5	90
#12	2+	5,5	363
#13	2+	5,5	208
#14	2+	5,5	219
#15	2+	5,5	252
#16	2+	5,5	363
#17	2+	5,5	186
#18	2+	5,5	429
#19	2+	5,5	208
#20	1+	2,5	125
#21	1+	2,5	72
#22	1+	2,5	160
#23	2+	5,5	231
#24	3+	9	845
#25	2+	5,5	363
#26	2+	5,5	373
#27	1+	2,5	77
#28	2+	5,5	297
#29	1+	2,5	83
#30	1+	2,5	72
#31	2+	5,5	274
#32	1+	2,5	158
#33	1+	2,5	40
#34	1+	2,5	160
#35	3+	9	1512
#36	1+	2,5	70
#37	1+	2,5	95
#38	1+	2,5	95
#39	1+	2,5	140
#40	1+	2,5	100
#41	1+	2,5	100
#42	1+	2,5	87
#43	1+	2,5	145
#44	1+	2,5	145
#45	1+	2,5	102
#46	1+	2,5	105
#47	2+	5,5	384
#48	1+	2,5	140
#49	2+	5,5	528
#50	1+	2,5	125
#51	1+	2,5	110
#52	1+	2,5	110
#53	1+	2,5	110
#54	2+	5,5	780
#55	1+	2,5	115
#56	1+	2,5	460

#57	1+	2,5	205
#58	1+	2,5	90
#59	2+	5,5	264
#60	1+	2,5	220
#61	2+	5,5	384
#62	1+	2,5	180
#63	1+	2,5	225
#64	1+	2,5	145
#65	1+	2,5	128
#66	1+	2,5	275
#67	1+	2,5	130
#68	1+	2,5	130
#69	1+	2,5	255
#70	1+	2,5	115
#71	1+	2,5	85
#72	3+	9	629
#73	1+	2,5	135
#74	1+	2,5	137
#75	1+	2,5	120
#76	1+	2,5	140
#77	2+	5,5	307
#78	2+	5,5	693
#79	1+	2,5	185
#80	1+	2,5	145
#81	1+	2,5	95
#82	1+	2,5	95
#83	1+	2,5	95
#84	1+	2,5	190
#85	2+	5,5	318
#86	3+	9	1925
#87	1+	2,5	265
#88	1+	2,5	68
#89	2+	5,5	1308
#90	1+	2,5	152
#91	1+	2,5	155
#92	1+	2,5	100
#93	1+	2,5	70
#94	1+	2,5	85
#95	2+	5,5	769
#96	1+	2,5	205
#97	1+	2,5	160
#98	1+	2,5	140
#99	1+	2,5	72
#100	2+	5,5	429
#101	1+	2,5	165
#102	1+	2,5	165
#103	1+	2,5	147
#104	1+	2,5	75
#105	1+	2,5	170
#106	2+	5,5	297
#107	1+	2,5	130
#108	2+	5,5	302
#109	2+	5,5	637
#110	1+	2,5	155
#111	1+	2,5	80
#112	1+	2,5	80
#113	1+	2,5	180
#114	1+	2,5	180
#115	2+	5,5	879
#116	1+	2,5	185

#117	1+	2,5	185
#118	1+	2,5	100
#119	1+	2,5	83
#120	1+	2,5	120
#121	1+	2,5	188
#122	1+	2,5	190
#123	1+	2,5	190
#124	1+	2,5	190
#125	1+	2,5	85
#126	1+	2,5	125
#127	1+	2,5	170
#128	1+	2,5	195
#129	1+	2,5	105
#130	1+	2,5	87
#131	1+	2,5	200
#132	1+	2,5	175
#133	2+	5,5	736
#134	1+	2,5	130
#135	1+	2,5	180
#136	1+	2,5	75
#137	2+	5,5	1799
#138	1+	2,5	210
#139	1+	2,5	135
#140	1+	2,5	185
#141	1+	2,5	95
#142	1+	2,5	95
#143	1+	2,5	160
#144	2+	5,5	776
#145	1+	2,5	115
#146	2+	5,5	1033
#147	1+	2,5	215
#148	1+	2,5	215
#149	1+	2,5	220
#150	2+	5,5	934
#151	1+	2,5	320
#152	1+	2,5	100
#153	1+	2,5	100
#154	1+	2,5	225
#155	1+	2,5	225
#156	1+	2,5	197
#157	1+	2,5	230
#158	1+	2,5	410
#159	2+	5,5	604
#160	2+	5,5	978
#161	1+	2,5	335
#162	1+	2,5	233
#163	2+	5,5	1143
#164	2+	5,5	736
#165	1+	2,5	515
#166	1+	2,5	180
#167	1+	2,5	240
#168	1+	2,5	245
#169	1+	2,5	245
#170	2+	5,5	759
#171	1+	2,5	110
#172	1+	2,5	110
#173	2+	5,5	1539
#174	1+	2,5	160
#175	1+	2,5	160
#176	1+	2,5	160

#177	1+	2,5	250
#178	1+	2,5	250
#179	1+	2,5	90
#180	2+	5,5	1231
#181	1+	2,5	165
#182	1+	2,5	115
#183	1+	2,5	295
#184	1+	2,5	335
#185	1+	2,5	230
#186	1+	2,5	170
#187	1+	2,5	385
#188	1+	2,5	525
#189	1+	2,5	173
#190	1+	2,5	173
#191	1+	2,5	270
#192	1+	2,5	120
#193	1+	2,5	120
#194	1+	2,5	120
#195	1+	2,5	270
#196	1+	2,5	270
#197	1+	2,5	175
#198	1+	2,5	275
#199	1+	2,5	207
#200	1+	2,5	280
#201	1+	2,5	125
#202	1+	2,5	125
#203	1+	2,5	323
#204	1+	2,5	285
#205	1+	2,5	285
#206	1+	2,5	290
#207	1+	2,5	290
#208	1+	2,5	290
#209	1+	2,5	627
#210	1+	2,5	293
#211	1+	2,5	255
#212	1+	2,5	295
#213	1+	2,5	338
#214	1+	2,5	132
#215	1+	2,5	160
#216	1+	2,5	260
#217	1+	2,5	260
#218	1+	2,5	85
#219	2+	5,5	780
#220	1+	2,5	195
#221	1+	2,5	137
#222	1+	2,5	310
#223	1+	2,5	200
#224	1+	2,5	315
#225	1+	2,5	90
#226	1+	2,5	320
#227	1+	2,5	205
#228	1+	2,5	365
#229	2+	5,5	703
#230	1+	2,5	210
#231	1+	2,5	210
#232	1+	2,5	330
#233	1+	2,5	120
#234	1+	2,5	180
#235	1+	2,5	233
#236	1+	2,5	215

#237	1+	2,5	293
#238	1+	2,5	185
#239	1+	2,5	125
#240	1+	2,5	350
#241	1+	2,5	355
#242	1+	2,5	160
#243	1+	2,5	160
#244	1+	2,5	160
#245	1+	2,5	160
#246	1+	2,5	195
#247	1+	2,5	195
#248	1+	2,5	365
#249	1+	2,5	235
#250	1+	2,5	325
#251	1+	2,5	240
#252	1+	2,5	375
#253	1+	2,5	240
#254	3+	9	8694
#255	1+	2,5	205
#256	1+	2,5	205
#257	1+	2,5	140
#258	1+	2,5	250
#259	1+	2,5	180
#260	1+	2,5	180
#261	1+	2,5	260
#262	1+	2,5	265
#263	1+	2,5	265
#264	1+	2,5	415
#265	1+	2,5	185
#266	1+	2,5	185
#267	1+	2,5	150
#268	1+	2,5	150
#269	1+	2,5	230
#270	1+	2,5	190
#271	1+	2,5	930
#272	1+	2,5	255
#273	1+	2,5	435
#274	1+	2,5	110
#275	1+	2,5	440
#276	1+	2,5	160
#277	1+	2,5	240
#278	1+	2,5	240
#279	1+	2,5	200
#280	1+	2,5	450
#281	1+	2,5	205
#282	1+	2,5	75
#283	1+	2,5	300
#284	1+	2,5	353
#285	1+	2,5	210
#286	1+	2,5	475
#287	1+	2,5	475
#288	1+	2,5	310
#289	1+	2,5	370
#290	1+	2,5	220
#291	1+	2,5	145
#292	1+	2,5	275
#293	1+	2,5	445
#294	1+	2,5	330
#295	1+	2,5	522
#296	1+	2,5	190

#297	1+	2,5	190
#298	1+	2,5	85
#299	1+	2,5	236
#300	1+	2,5	400
#301	1+	2,5	240
#302	1+	2,5	155
#303	2+	5,5	3409
#304	1+	2,5	245
#305	2+	5,5	1188
#306	1+	2,5	200
#307	1+	2,5	250
#308	1+	2,5	725
#309	1+	2,5	260
#310	1+	2,5	260
#311	1+	2,5	215
#312	1+	2,5	390
#313	1+	2,5	330
#314	1+	2,5	275
#315	1+	2,5	795
#316	1+	2,5	335
#317	1+	2,5	280
#318	1+	2,5	280
#319	1+	2,5	180
#320	1+	2,5	482
#321	1+	2,5	665
#322	1+	2,5	190
#323	1+	2,5	245
#324	2+	5,5	2122
#325	1+	2,5	195
#326	1+	2,5	440
#327	1+	2,5	690
#328	1+	2,5	260
#329	1+	2,5	325
#330	1+	2,5	400
#331	1+	2,5	335
#332	1+	2,5	215
#333	1+	2,5	765
#334	1+	2,5	425
#335	1+	2,5	285
#336	1+	2,5	792
#337	1+	2,5	610
#338	1+	2,5	305
#339	1+	2,5	385
#340	1+	2,5	255
#341	1+	2,5	405
#342	1+	2,5	685
#343	1+	2,5	920
#344	1+	2,5	440
#345	1+	2,5	1450
#346	1+	2,5	680
#347	1+	2,5	485
#348	1+	2,5	700
#349	1+	2,5	395
#350	1+	2,5	490
#351	1+	2,5	1265
#352	1+	2,5	495
#353	1+	2,5	720
#354	1+	2,5	505
#355	1+	2,5	290
#356	1+	2,5	335

#357	1+	2,5	1035
#358	1+	2,5	350
#359	1+	2,5	1430
#360	1+	2,5	800
#361	1+	2,5	825
#362	1+	2,5	800
#363	1+	2,5	2625
#364	1+	2,5	1028
#365	1+	2,5	2400
